# Disrupting rhythms in *Plasmodium chabaudi*: costs accrue quickly and independently of how infections are initiated

**DOI:** 10.1186/1475-2875-12-372

**Published:** 2013-10-26

**Authors:** Aidan J O’Donnell, Nicole Mideo, Sarah E Reece

**Affiliations:** 1 Institutes of Evolution, Immunology and Infection Research, University of Edinburgh, Edinburgh, UK; 2 Department of Ecology and Evolutionary Biology, University of Toronto, Toronto, Canada; 3 Centre for Immunity, Infection and Evolution, University of Edinburgh, Edinburgh, UK

**Keywords:** Developmental rhythms, Circadian clock, Fitness, Malaria, Ring stage, Trophozoite, Intravenous, Intraperitoneal, Synchronicity, Phase-shift

## Abstract

**Background:**

In the blood, the synchronous malaria parasite, *Plasmodium chabaudi*, exhibits a cell-cycle rhythm of approximately 24 hours in which transitions between developmental stages occur at particular times of day in the rodent host. Previous experiments reveal that when the timing of the parasite’s cell-cycle rhythm is perturbed relative to the circadian rhythm of the host, parasites suffer a (~50%) reduction in asexual stages and gametocytes. Why it matters for parasites to have developmental schedules in synchronization with the host’s rhythm is unknown. The experiment presented here investigates this issue by: (a) validating that the performance of *P. chabaudi* is negatively affected by mismatch to the host circadian rhythm; (b) testing whether the effect of mismatch depends on the route of infection or the developmental stage of inoculated parasites; and, (c) examining whether the costs of mismatch are due to challenges encountered upon initial infection and/or due to ongoing circadian host processes operating during infection.

**Methods:**

The experiment simultaneously perturbed the time of day infections were initiated, the stage of parasite inoculated, and the route of infection. The performance of parasites during the growth phase of infections was compared across the cross-factored treatment groups (i e, all combinations of treatments were represented).

**Results:**

The data show that mismatch to host rhythms is costly for parasites, reveal that this phenomenon does not depend on the developmental stage of parasites nor the route of infection, and suggest that processes operating at the initial stages of infection are responsible for the costs of mismatch. Furthermore, mismatched parasites are less virulent, in that they cause less anaemia to their hosts.

**Conclusion:**

It is beneficial for parasites to be in synchronization with their host’s rhythm, regardless of the route of infection or the parasite stage inoculated. Given that arrested cell-cycle development (quiescence) is implicated in tolerance to drugs, understanding how parasite schedules are established and maintained in the blood is important.

## Background

**Figure 1:**
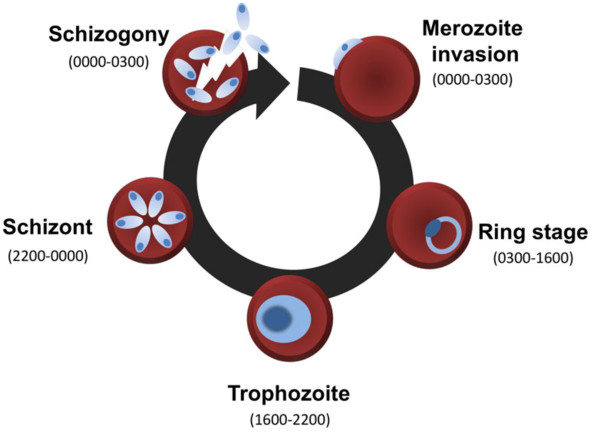
**The cell cycle of *Plasmodium.*** For *Plasmodium chabaudi*, progressing through these developmental stages takes 24 hours. Approximate host circadian times are given in parentheses.

Biological rhythms are ubiquitous in taxa spanning bacteria to vertebrates, eliciting periodicity in a multitude of biological processes and behaviours. Accurately matching biological rhythms to the daily rotation of the Earth appears to be important for competitive ability (cyanobacteria and plants) [1,2], growth rate (insects) [3], and reproductive success (plants and insects) [4-7]. In the blood, the synchronous malaria parasite, *Plasmodium chabaudi*, exhibits a cell-cycle rhythm of approximately 24 hours in which transitions between developmental stages occur at particular times of day in the rodent host (Figure 1) [8]. Such synchronous development has been documented in many species of malaria parasite, including those that infect humans (reviewed in [9]). Perturbing the timing of the *P. chabaudi* cell cycle relative to the host’s circadian rhythm causes a two-fold reduction in the densities of both asexual and sexual transmission stages [10]. This has implications for parasite fitness because low densities of asexual stages make parasites vulnerable to clearance by the immune system and poor competitors in mixed infections and (in general) sexual stage density correlates positively with the success of transmission to mosquitoes [11-15]. While the net fitness costs for parasites of perturbing their coordination with the biological rhythm of the host are apparent, the processes that reduce asexual and gametocyte densities during perturbation are unknown.

The reduced performance of schedule mismatched parasites observed in [10] does not reveal whether coordination between parasite cell-cycle progression and the host circadian rhythm is controlled by parasites or hosts or both. This remains an important route of future investigation which will be facilitated by better characterisation of the costs of mismatch. This includes determining when the costs of mismatch materialize: are the costs of mismatch a result of time-of-day-dependent challenges encountered upon initial infection and/or challenges experienced continuously throughout infections? Though the cell cycles of mismatched parasites eventually adjust to be in synchrony with the host circadian rhythm [16], prior to this, parasites in each cell cycle may enter a particularly vulnerable stage in their development at a time when circadian aspects of the within-host environment are least favourable. For example, parasite developmental stages may vary in their sensitivity to peaks in the rhythms of innate immune defences in the blood/spleen or the nutritional requirements of different stages may not be met at certain times of day. These time-of-day dependent challenges could affect parasites as they enter the host (if, for instance, low densities of parasites are particularly vulnerable, or these processes operate at the site of infection) and/or during every cycle as infections progress. Distinguishing between these alternatives is non-trivial, not least because even small costs that arise during initial establishment will propagate and magnify with successive rounds of replication, resulting in reduced overall performance. However, a clear prediction is that if mismatch causes costs in the initial phase of infections there will be fewer parasites appearing in the blood and if costs are due to ongoing processes, there will be differences in multiplication rate throughout infections.

This study asks when the costs of mismatch appear and also addresses two issues raised by the results of [10]. First, the route of infection in [10] was via intraperitoneal injection, either in the host’s morning or evening. If circadian host processes play a role in the establishment phase of experimental infections, then mismatched parasites may have performed poorly because of time-of-day dependent challenges experienced in the peritoneal cavity. For example, given the circadian periodicity of macrophage activity [17], parasites injected in the evening were likely to encounter peritoneal macrophages in the peak of their protective activity. In this case, the costs of mismatch would arise in the initial stage of infections, but since the peritoneal cavity is not the natural mode of infection, nor an environment blood stage malaria parasites naturally encounter, the effects reported in [10] may not be biologically relevant. Second, the same parasite stage (rings) was used to establish the infections in [10], but parasite cell-cycle stages may differ in their sensitivity to time-of-day-dependent challenges. For example, different stages may be more sensitive to peritoneal macrophages at the peak of their activity. In this case, the costs of mismatch may be due to an interaction between host time of day and the parasite developmental stage injected. Characterising how the of costs of mismatch are affected by timing, route of infection, and parasite developmental stage will help to identify the mechanisms underpinning parasite schedules and could provide new insight for control. For example, drugs given at certain times of day could be more effective through synergy with host circadian immune responses or by targeting parasites at their most vulnerable cell-cycle stage.

The aims of the experiment reported here were to validate that the performance of *P. chabaudi* is negatively affected by mismatch to the host circadian rhythm, test whether the costs of mismatch are influenced by the route of infection or the developmental stage of inoculated parasites, and to examine whether the costs of mismatch are due to challenges encountered upon initial infection or to processes operating throughout the infection. This required simultaneously perturbing the stage of parasite inoculated, host time of day, and route of infection, and measuring parasite performance at the start and during infections. The impact to the host is also considered, using red blood cell loss as a measure of parasite virulence [11,18,19]. The results confirm that mismatch to host rhythms is costly for parasites, reveal that this phenomena does not depend on the developmental stage of parasites nor the route of infection (i e, it is not simply a consequence of challenges experienced in the peritoneal cavity), and suggest that processes operating at the initial stages of infection are responsible for the costs of mismatch.

## Methods

### Parasites and hosts

Hosts were ten to 12-week old MF1 male mice housed at 21°C with *ad lib* food and drinking water supplemented with 0.05% para-aminobenzoic acid (to support parasite growth). The synchronous *P. chabaudi* clone (AJ) was used [10]. Manipulating the circadian rhythms of hosts was achieved by housing mice in two rooms, each maintained on a 12-hour light: dark cycle that differed only in the timing of lights-on. In the “standard schedule” room, lights were on during the day (lights on: 07.30; lights off: 19.30); in the “light reversed” room, lights were on during the night (lights on 19.30; lights off: 07.30). All mice in the experiment were allowed to acclimatize to their respective light: dark schedule for two weeks before infection. This allowed mice to entrain to their schedule, as previous work has demonstrated this occurs within seven days [20]. Prior to infection it was verified that the mice behaved as expected for their light: dark schedule (e.g., were active during the dark period and inactive when lights were on). In each room, a donor host was infected with 1 × 10^6^*P. chabaudi* (clone AJ) parasitized red blood cells (RBCs) to provide parasites to initiate experimental infections. All procedures were carried out in accordance with the UK Home Office regulations (Animals Scientific Procedures Act 1986; 60/4121) and approved by the ethical review panel at Edinburgh University.

### Experimental design

**Figure 2:**
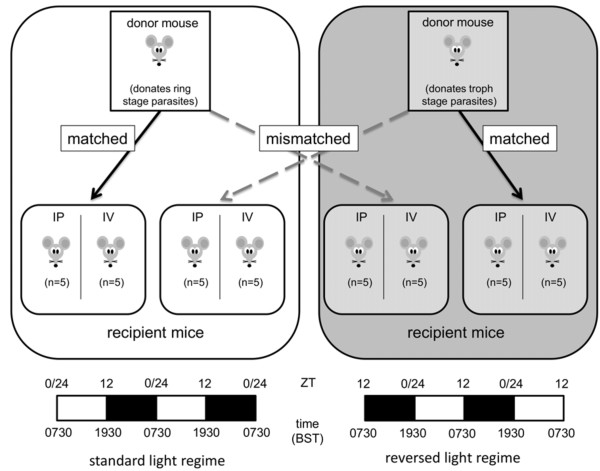
**Experimental design.** Arrows indicate the transfer of parasites to recipient hosts (eight groups of five mice) within and between two rooms with opposite light schedules. Parasites remaining in the same room are matched to host rhythms and act as controls. Parasites transferred between rooms to hosts that are on the opposite rhythm to the parasite donor were temporally mismatched, analogous to jetlag. At the time of transfer, parasites originating from the standard light regime donor were at ring stage and those originating from the reversed light regime donor were at trophozoite stage and infections were initiated via either intraperitoneal (IP) or intravenous (IV) routes. Dark and light bars indicate lights on/off status throughout a 48-hour period. Zeitgeber time (ZT) is displayed above the bars; ZT 0/24, the time of lights on and ZT 12, time of lights off.

Mice for the experiment were housed in groups of five and a total of 40 were used (n = 5 infections per treatment group). All experimental infections were initiated in the morning (11.00 GMT) (Figure 2). This permitted simultaneous infections using two different parasite stages. Donor infections originating from the “standard schedule” room were used to *simultaneously* infect mice in the “standard schedule” room and the “light reversed room” with ring-stage parasites (hereafter, rings). The same procedure was repeated for parasites from the “light-reversed” room to *simultaneously* initiate infections in each room with late trophozoite-stage parasites (hereafter, trophozoites). This created two groups of infections in which parasite stage and host circadian rhythm were *matched* (e.g., mice in their morning received rings, and mice in their evening received trophozoites) and *mismatched* (e.g., mice in their morning received trophozoites, and mice in their evening received rings). That parasites were at the required stage for initiating infections was verified via blood smear at the time of harvesting. Parasites were administered either via intraperitoneal injection (IP) or intravenous injection (IV), at a dose of 1 × 10^6^ parasitized RBC. This created a total of eight treatments (Figure 2) to include all combinations (cross-factoring) of route of infection (IP or IV), parasite stage (ring or trophozoite), and parasite and host rhythms (schedule matched or mismatched).

### Data collection

All mice were sampled daily, in the morning at 09.00 GMT (e.g., beginning at 22 hours post infection), during the growth phase of *P. chabaudi* AJ infections (until day 7 post infection (pi) when starting with 10^6^ parasitized RBC [21]). This timing is consistent with previous work [10] and is prior to any adjustment of the schedule of mismatched parasites to become synchronised with the host rhythm [10,16,22-24]. At each sampling point, 5 μl blood samples were taken to quantify total parasite densities using quantitative PCR (qPCR). DNA was extracted using the ABI Prism 6100® according to the manufacturer’s protocol. Total parasite densities were obtained using primers based on the gametocyte-expressed gene PC302249.00.0 [25]. RBC densities were measured on days 1, 3 and 7 pi using flow cytometry (Beckman Coulter).

### Data analysis

R version 2.6.1 [26] was used for all analyses. General linear models were used to test how the perturbations of the route of infection, parasite stage, and co-ordination of parasite and host rhythms affected (i) the ability of parasites to establish infections (days 1 and 2 pi) and, (ii) their overall performance to the peak of infections (cumulative density between days 1–7). General linear mixed effects models were used to examine whether replication rate was affected by mismatch of host and parasite rhythms. This required fitting mouse identity as random effect to control for the non-independence of multiple data points from each infection [27]. Maximal models contained all main effects and interactions, and models were minimized using stepwise deletion until only significant terms remained. Parasite multiplication rate was calculated as the number of parasites observed on day *t* + 1 divided by the number on the previous day (*t*).

## Results

**Figure 3:**
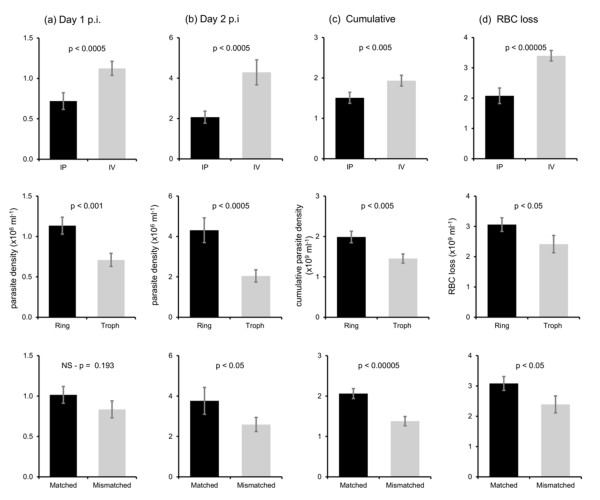
**Parasites performed better when injected via intravenous, at ring-stage, and matched to host rhythms, resulting in greater virulence.** Total parasite densities of infections **(a)** on day 1 post-infection, **(b)** on day 2 post infection, and **(c)** summed from day 1 to day 7 pi (cumulative density); **(d)** overall loss of RBCs during the pre-peak phase. Bars show mean (±se) densities of parasites or RBC with (a) n = 39 infections per group (b-d) n = 40 per group. The top row compares the route of infection either by IP (intraperitoneal injection, black bars) or IV (intravenous injection, grey bars). The middle row compares the parasite stage used to initiate the infections, with rings (black bars) or trophozoites (grey bars). The bottom row compares parasites on the same (matched, black bars) or perturbed (mismatched, grey bars) schedule as the host.

**Figure 4:**
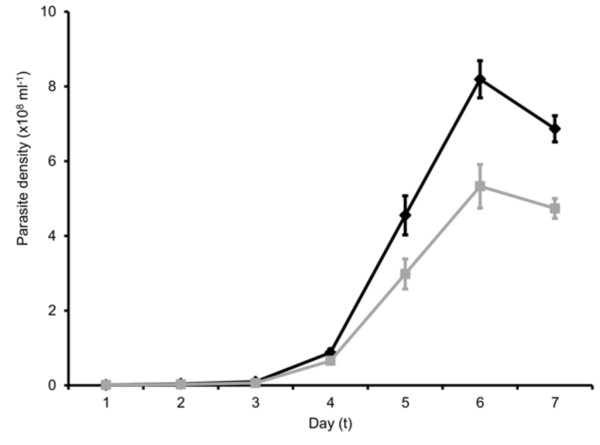
**Matched parasites performed better than mismatched parasites throughout the pre-peak phase of the infection.** Temporal dynamics of all infections (route and stage treatments combined) followed from day 1 to day 7 pi. The mean (±se) densities of matched (black lines) and mistmatched (grey lines) infections are plotted.

The route of infection, parasite stage, and mismatch between host and parasite schedules all had significant effects on parasite densities (Figure 3). Infections via IV had significantly higher densities on day 1 (F_(1, 36)_ = 14.70; P <0.001) and 2 (F_(1, 36)_ =15.50; P <0.001) pi, and this was maintained throughout the pre-peak phase of the infection (as demonstrated by cumulative parasites densities; F_(1, 36)_ = 10.09; P = 0.003). Infections initiated with rings performed significantly better than infections initiated with trophozoites on day 1 (F_(1, 36)_ = 12.75; P = 0.001) and 2 (F_(1, 36)_ = 16.10; P <0.001) pi, and throughout the pre-peak phase of the infection (cumulative parasites densities; F_(1, 36)_ = 15.89; P <0.001). On day 1 post-infection, the densities of matched and mismatched parasite densities did not differ significantly (F_(1, 36)_ = 1.76; P = 0.193) though the densities of mismatched parasites tended to be lower. By day 2, however, matched parasites performed significantly better than mismatched parasites (F_(1, 36)_ = 4.33; P = 0.045) and this pattern was maintained throughout the pre-peak phase (cumulative parasites densities; F_(1, 36)_ = 26.01; P <0.001), as can be seen in the temporal dynamics (Figure 4). The means (±se) for the significant effects and R squared values for the minimal models are given in Table 1.

**Table 1:** Effects of experimental treatments on parasite densities (means ± se)

	**Host and parasite schedules**		**Route of infection**		**Stage injected**		**Rsq**
	***Matched***	***Mismatched***	***IP***	***IV***	***Rings***	***Trophozoites***	
Day 1 pi	1.02 ± 0.01	0.84 ± 0.01	0.72 ± 0.10	1.12 ± 0.09	1.13 ± 0.10	0.71 ± 0.09	0.433
Day 2 pi	3.76 ± 0.67	2.59 ± 0.35	2.07 ± 0.30	4.29 ± 0.62	4.31 ± 0.61	2.05 ± 0.30	0.458
Cumulative	2.06 ± 0.12	1.38 ± 0.12	1.51 ± 0.14	1.93 ± 0.13	1.99 ± 0.14	1.45 ± 0.11	0.591

Rsq values for minimal models are included. Note, there was no significant difference between matched or mismatched parasites on day 1 pi. Day 1 and 2 pi are × 10^6^ /mL and cumulative densities are × 10^9^ /mL.

**Figure 5:**
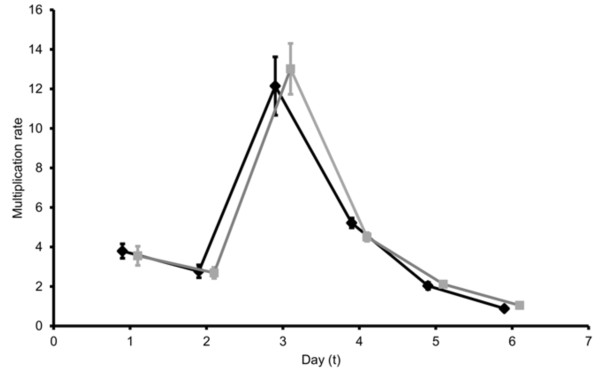
**Multiplication rate (number of progeny produced per parasite).** The means (±se) for matched (black lines) and mismatched (grey lines) infections are plotted for each cycle of replication (data plotted on the x-axis are offset for clarity), calculated as the number of parasites observed on day *t* + 1 divided by the number on the previous day (*t*). For example, data plotted on day 1 represent the multiplier between day 1 to day 2.

There were no significant interactions between host-parasite schedules and the route of infection or parasite stage (all P > 0.60). This reveals that mismatch has equal effects on parasites administered IP and IV, and on ring and trophozoite stages. This allows treatment groups to be combined to directly compare matched with mismatched parasites to examine whether the costs of mismatch stem from processes that operate during infections to constrain replication (Figure 5). The number of progeny produced by each parasite (multiplication rate) varies during infections (χ^2^_5_ = 263.31; P <0.001) but does not differ significantly between matched and mismatched parasites, for all cell cycles examined (Schedule: χ^2^_1_ = 0.01; P = 0.964; day by schedule interaction: χ^2^_5_ = 1.86; P = 0.868). This result, taken together with the significant difference in densities appearing by day 2 pi suggests that circadian processes operating in the initial phase of infection reduce parasite number and this initial difference is propagated throughout infections to result in significant costs of mismatch with the host rhythm.

It is easy to show algebraically that any small difference in initial parasite densities between matched and mismatched parasites will increase at a rate proportional to the multiplication rate, even when each parasite produces the same number of progeny per cell cycle. If the initial densities of matched and mismatched parasites are *p* and *p + ϵ,* respectively, and the multiplication rate of all parasites is *r*, then after *t* days (rounds of replication) the density of matched and mismatched parasites will be *r*^*t*^*p* and *r*^*t*^(*p* + *ϵ*) and the difference in densities between matched and mismatched infections will have increased by a factor of *r*^*t*^ (i e, from *ϵ* to *r*^*t*^*ϵ*). Even if multiplication rates change over time (i e, *r* changes over time, as is the case; Figure 5), as long as it is greater than 1, the difference between matched and mismatched parasite densities will increase as infections progress.

Finally, hosts lost RBCs throughout the pre-peak phase of the infection and the patterns mirrored parasite performance. Hosts infected via IV lost significantly more RBC (i e, had greater anaemia) than via IP (F_(1, 36)_ = 22.49; P <0.001), hosts receiving ring-stage parasites lost more RBCs than those receiving trophozoites (F_(1, 36)_ = 5.36; P = 0.026), and matched parasites caused greater anaemia than mismatched parasites (F_(1, 36)_ = 6.13; P = 0.018). Again there were no significant interactions (all P > 0.29) between schedule, route, and stage affecting RBC loss.

## Discussion

This experiment involved the simultaneous perturbation of coordination between host and parasite schedules, the stage of parasite inoculated, and the route of infection. The data confirm that mismatch to host rhythms is costly for *P. chabaudi* parasites and reveal that this phenomena does not depend on the developmental stage inoculated nor the route of infection. Coupled with previous work [10], the data demonstrate that a phase-shift of between nine to 12 hours is detrimental for parasites. Moreover, further analyses reject the hypothesis that the costs of mismatch are due to processes that reduce the multiplication rate of parasites throughout infections, but instead, suggest that processes operating when parasites are establishing a blood stage infection are responsible. The lack of impact of time-of-day effects throughout infections cannot be explained by parasite schedules quickly adjusting to become synchronised with the host circadian rhythm. By staging parasites in blood smears we verified that, 3 days after inoculation, parasites were maintaining their original developmental schedule (data not shown), and previous work suggests that any adjustment takes at least 7 days [10,16,22-24].

The experiment also revealed that, as expected, ring stage parasites are more successful in establishing infections (which is presumably why, conventionally, ring stages initiate experimental infections) than trophozoite stages and both stages benefit from being injected straight into the blood stream rather than having to negotiate their way from the peritoneal cavity to the blood (by an as yet unknown mechanism). The effects of parasite stage and route of infection were apparent by 1 pi. Finally, the negative effects of schedule mismatch on parasite performance have consequences for virulence because hosts receiving mismatched parasites suffer less anaemia than those infected with matched parasites.

What host circadian processes could act on parasites in the initial stage of infection only? Given that the cost of mismatch is independent of the route of infection and that it may manifest between day 1–2 pi (when the IP-injected parasites have appeared in the blood) processes operating in the bloodstream are likely responsible. An intriguing possibility is that mismatch between the recipient host and the rhythm of the donor RBC, rather than the parasites themselves, generates an early cost. Recent work has demonstrated that RBCs have their own circadian rhythms, driven by the redox state of the cell [28,29]. If the mismatch between the donor RBC’s state and the recipient host’s rhythm leads to these cells being preferentially filtered by the spleen or targeted by housekeeping immune responses, then this would generate an early cost for mismatched parasites. However, many components of the mammalian immune system in the blood and spleen exhibit circadian periodicity [17,30-37], so if these are involved in clearing unwanted RBC we would not expect to see costs in both mismatched treatment (since these processes are unlikely to be at their peak activity in both the host's morning and night). However, whether parasitised RBC maintain a normal redox rhythm and hosts can discriminate the RBC redox state of either the infected and/or uninfected RBC present in the inocula, regardless of whether they are injected in the morning or evening, is unknown. If such mechanisms exist, the progeny of parasites that survived the first day in the bloodstream would infect a host RBC on the correct schedule, and thus would not subsequently suffer from the same cost.

Another possibility is that dead parasites/RBC in the inocula – but not the ongoing live infection – provide a transient extra stimulation for innate effectors with circadian schedules. Both this and the RBC redox state explanation are unconvincing because their effects are likely to be apparent on day 1 pi. A more plausible scenario is that parasites must exceed a density threshold to activate early innate responses (e g, a density that is achieved after day 1 in this experiment) and that these responses can be overwhelmed at high parasite densities [38]. This would make the cost of mismatch greatest, and perhaps only apparent, at intermediate densities. More work is required to determine whether costs of mismatch were not apparent on day 1 pi due to lack of statistical power. Statistically detecting a small effect requires a large sample size and a multivariate power analysis reveals that with 20 infections per group, as for this experiment, the chance of detecting a significant effect on day 1 pi (given the observed means and variances) is 73%. Therefore, repeating the experiment with larger sample sizes, reducing the variation in density estimates across infections (e.g., by assaying multiple samples per infection each day), and including other infective doses will enable more thorough investigation of the timing of the costs of mismatch.

That the cost of schedule mismatch is not influenced by either the route of infection (IP or IV) or parasite stage (ring or trophozoite) is unexpected. Macrophages line the peritoneal cavity and have an autonomous 24-hour clock that regulates phagocytosis and the rhythmic secretion of TNF and IL-6 in response to infection, with peak activity late in the day [17,35,37]. Parasites administered via IP in the evening were therefore expected to experience a harsher environment than parasites inoculated IP in the morning. Furthermore, late-stage parasites are thought to be more susceptible to stress than rings, as suggested for fever (e.g., heat shock disproportionately kills parasites in the latter half of the cell cycle [39,40]).

Therefore, trophozoite-stage parasites were expected to be more vulnerable to time-of-day effects compared to infections initiated with rings. If such stressors included active macrophages then inoculation of trophozoites in the evening via IP would result in the poorest performing infections. This is not the case because trophozoites are not disproportionately disadvantaged by time, nor route, of infection.

## Conclusions

It is beneficial for parasites to be in synchrony with their host’s rhythm, regardless of the route of infection or the parasite stage inoculated. The data presented here suggest mismatch impacts on the ability of parasites to establish infections, but not on their ability to multiply, and that the reduction in ‘starting number’ has a magnifying effect on density as infections progress. While the coordination between parasites and host rhythms is apparent, whether this is actively achieved by the parasite or passively established by host rhythms remains unknown. Because hosts infected by mismatched parasites experience less severe anaemia, hosts would benefit by causing parasites to become mismatched. Hosts do not appear to do this, suggesting that hosts are not in control of parasite schedules, or that host rhythms are unavoidably responsible for parasite schedules. How parasites benefit from synchronisation with the host, and why this is particularly important at the start of infections, also remains unknown. The answers to these questions may be revealed by identifying whether parasite stages differ in their vulnerability to circadian innate effectors, if parasites have resource requirements that are only met at certain times of day, how these processes are affected by parasite density, and whether the costs of mismatch vary across different durations of time shift. Given that arrested cell-cycle development (quiescence) is implicated in tolerance to drugs [41-45], understanding what governs these schedules as well as the costs and benefits of adjusting them is important.

## Competing interests

The authors declare that they have no competing interests.

## Authors’ contributions

AOD and SR conceived and designed the project; AOD carried out the experiment; all authors analysed the data and prepared the manuscript. All authors read and approved the final manuscript.
