# Erratum to: disrupting rhythms in *Plasmodium chabaudi*: costs accrue quickly and independently of how infections are initiated

**DOI:** 10.1186/1475-2875-13-503

**Published:** 2014-12-17

**Authors:** Aidan J O’Donnell, Nicole Mideo, Sarah E Reece

**Affiliations:** Institutes of Evolution, Immunology and Infection Research, University of Edinburgh, Edinburgh, UK; Department of Ecology and Evolutionary Biology, University of Toronto, Toronto, Canada; Centre for Immunity, Infection and Evolution, University of Edinburgh, Edinburgh, UK

## Correction

Some of the data in the article [1] were inadvertently mislabelled. Specifically, for infections initiated with trophozoite stage parasites, the schedule “matched” treatment group was incorrectly analysed as “mismatched” and vice-versa. The data have been re-analysed and the effects of perturbing the schedules of parasites relative to the host circadian rhythm are more complex than presented in the original paper. However, the differences between initiating infections with ring stages versus trophozoite stages, and via intraperitoneal injection or intravenous injection remain unchanged. The affected sections of the paper (data analysis method, results, discussion) have been re-written and new figures drawn. The authors apologize for any inconvenience or confusion that this may have caused.

### Data analysis

R version 2.6.1 (The R foundation for statistical computing; http://www.R-project.org; Vienna, Austria) was used for all analyses. General Linear Models were used to test how the perturbations of the route of infection, parasite stage, and co-ordination of parasite and host rhythms affected (i) the ability of parasites to establish infections (days 1 and 2 pi) and (ii) their overall performance to the peak of infections (cumulative density between days 1–7). Data for day 2 post-infection were log_10_ transformed to conform to the assumptions of normality. General linear mixed effects models were used to examine whether replication rate was affected by mismatch of host and parasite rhythms. This required fitting mouse identity as random effect to control for the non-independence of multiple data points from each infection [2]. Maximal models contained all main effects and interactions, and models were minimised using stepwise deletion until only significant terms remained.

## Results

The route of infection, parasite stage, and mismatch between host and parasite schedules all had significant effects on parasite densities (Figure 1, replaces Figure three). The influence of these factors varied across infections and explained between 42-59% (R^2^) of variation in parasite numbers.

**Figure 1:**
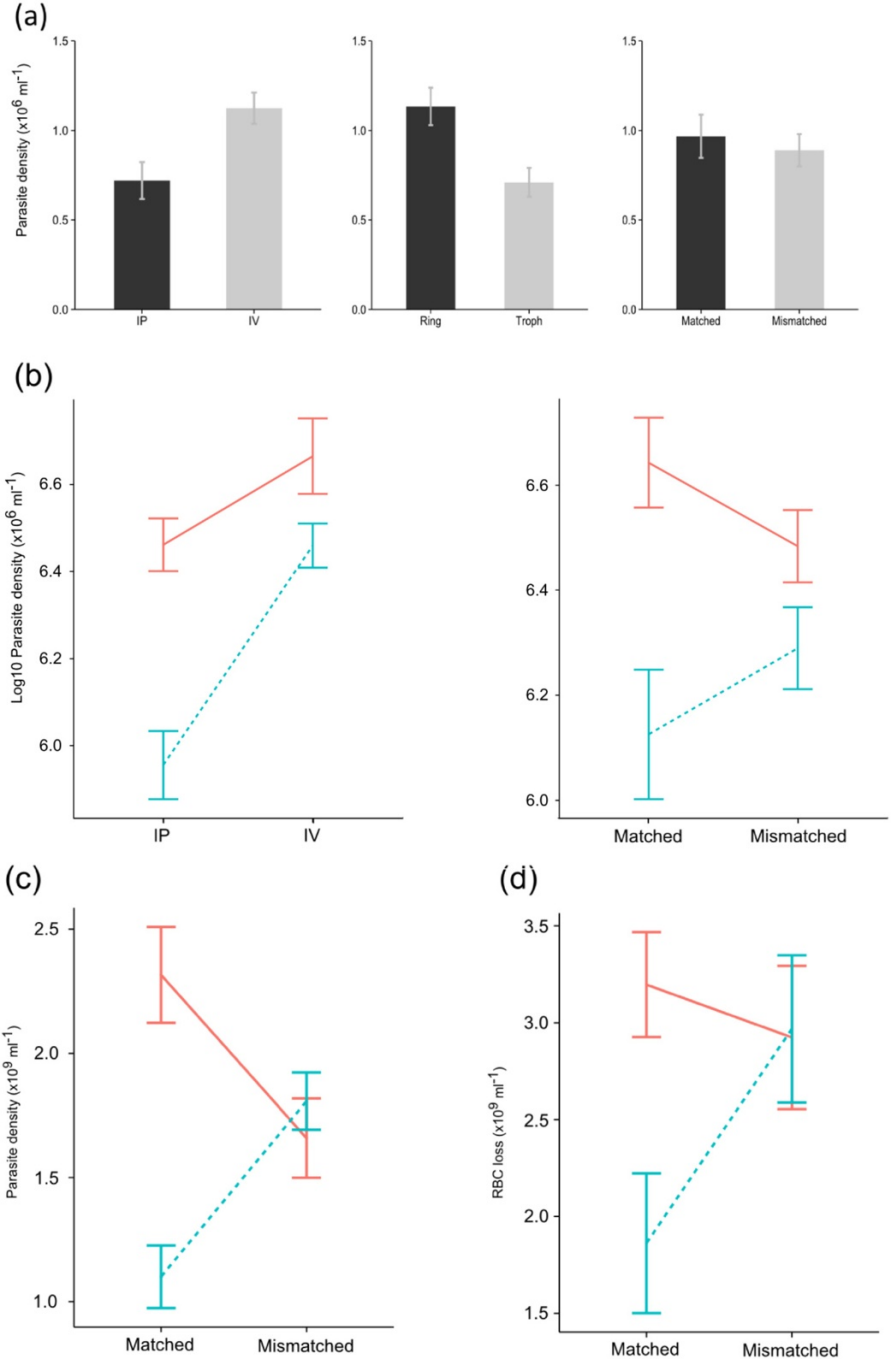
**From Day 2 the impact of mismatch varies based on which parasite stage initiated the infection. (a)** Parasite densities of infections on Day 1 post-infection. Bars show mean (±se) densities of parasites with n =39 infections. The left plot compares the route of infection either by IP (intraperitoneal injection, black bars) or IV (intravenous injection, grey bars). The middle plot compares the parasite stage used to initiate the infections, with rings (black bars) and trophozoites (grey bars). The right plot compares parasites on the same (matched, black bars) or perturbed (mismatched, grey bars) schedule as the host. Parasite stage (rings, solid lines; trophozoites, dotted lines) and whether parasites were matched or mismatched to the host schedule had significant effects on Day 2 post infection **(b)** and across the pre-peak phase **(c).** Mean (±se) densities are plotted (note for **(b)** the analysis required the data to be transformed). The mean (±se) amount of RBC lost hosts depended on the stage and schedule of parasites they were infected with **(d).** n =40 infections for **(b) – (d).**

On day 1 (Figure 1a), infections via IV had significantly higher densities than via IP (F_(1, 36)_ =12.90; P <0.001) and infections initiated with rings performed significantly better than infections initiated with trophozoites (F_(1, 36)_ =13.40; P <0.001; R^2^ = 0.42). However, the densities of matched and mismatched parasite densities did not differ significantly (F_(1, 36)_ =0.22; P =0.640). On Day 2 (Figure 1b), there were significant interactions between route of infection and parasite stage (F_(1, 34)_ =5.04; P =0.031) and between parasite schedule and parasite stage (F_(1, 34)_ =5.84; P =0.021; R^2^ = 0.52). Infections initiated with rings always had higher densities than infections initiated with trophozoites, and this difference was greatest when the route of infection was IP. Mismatch had a substantial negative effect on infections initiated with rings but not trophozoites (R^2^ = 0.52). These effects became more pronounced over the pre-peak phase of the infection (Figure 1c; R^2^ = 0.59): mismatch was costly (1.4 fold reduction) for infections initiated with rings but beneficial (1.6 fold increase) to those initiated with trophozoites (F_(1, 35)_ =5.84; P =0.021), and higher parasite densities were always observed in infections via IV compared to IP (F_(1, 35)_ =9.82; P =0.003).

Hosts lost RBCs throughout the pre-peak phase of the infection and the patterns mirrored parasite performance (Figure 1d; R^2^ = 0.52). Hosts infected via IV lost significantly more RBC (i e, had greater anaemia) than via IP (F_(1, 35)_ =22.32; P <0.001). Again, there was a significant interaction between schedule and stage (F_(1, 35)_ =6.35; P =0.016) in which hosts infected with matched trophozoites lost the least RBC.

The number of progeny produced by each parasite (multiplication rate) varied during infections (χ^2^_5_ = 263.32; P <0.001) but did not differ significantly between matched and mismatched parasites, for all replication cycles examined (Schedule: χ^2^_1_ = 0.302; P =0.582) (Figure 2, replaces Figure five). This result, taken together with the significant difference in densities appearing by day 2 pi suggests that circadian processes operating in the initial phase of infection affect parasite number in a stage-specific manner (benefit trophozoites and harm rings) and this initial difference is propagated throughout infections to result in significant effects of mismatch with the host rhythm.

**Figure 2:**
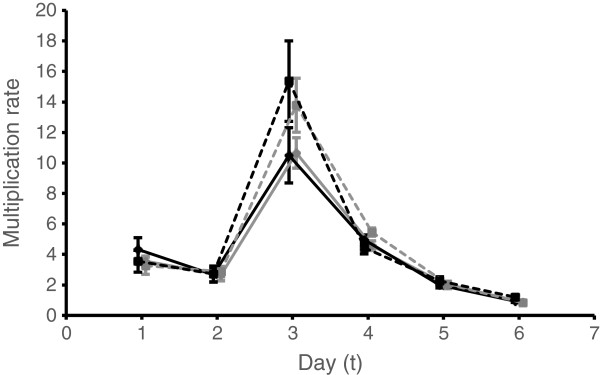
**Multiplication rate (number of progeny produced per parasite).** The means (±se) for matched (black lines) and mismatched (grey lines) infections initiated by rings (solid lines) and trophozoites (dotted lines) are plotted for each cycle of replication (the x-axis is offset for clarity), calculated as the number of parasites observed on day t +1 divided by the number on the previous day (t). For example, data plotted on day 1 represent the multiplier between day 1 to day 2.

It is easy to show algebraically that any small difference in parasite densities, such as the difference observed between matched and mismatched parasites by day 1 post infection, will increase at a rate proportional to the multiplication rate, even when each parasite produces the same number of progeny per cell cycle. If the initial densities of matched and mismatched parasites are *p* and *p + ϵ,* respectively, and the multiplication rate of all parasites is *r*, then after *t* days (rounds of replication) the density of matched and mismatched parasites will be *r*^*t*^*p* and *r*^*t*^(*p* + *ϵ*) and the difference in densities between matched and mismatched infections will have increased by a factor of *r*^*t*^ (i e, from *ϵ* to *r*^*t*^*ϵ*). Even if multiplication rates change over time (i e, *r* changes over time, as is the case; Figure 2), as long as it is greater than 1, the difference between matched and mismatched parasite densities will increase as infections progress.

## Discussion

This experiment involved the simultaneous perturbation of coordination between host and parasite schedules, the stage of parasite inoculated, and the route of infection. The data show that mismatch to host rhythms is costly for *P. chabaudi* parasites regardless of the route of infection, but reveal that this phenomena depends on the developmental stage inoculated. The experiment also revealed that, as expected, ring stage parasites are generally more successful in establishing infections than trophozoite stages (which is presumably why, conventionally, ring stages are used to initiate experimental infections) and both stages benefit from being injected straight into the blood stream rather than having to negotiate their way from the peritoneal cavity to the blood (by an as yet unknown mechanism). Finally, the interaction between co-ordination of parasite and host rhythms and parasite stage may have consequences for virulence because mice in infected with matched trophozoite stages suffer less anaemia than mice in the other treatment groups.

This experiment, coupled with previous work [3], confirm that a phase-shift of between nine to 12 hours is detrimental for ring stage parasites and unexpectedly reveal that phase-shift is beneficial for trophozoite stage parasites. Moreover, further analyses reject the hypothesis that the costs of mismatch are due to processes that reduce the multiplication rate of parasites throughout infections, but instead, suggest that processes operating when parasites are establishing a blood stage infection are responsible. The lack of impact of time-of-day effects throughout infections cannot be explained by parasite schedules quickly adjusting to become synchronised with the host circadian rhythm. Staging parasites in blood smears verified that 3 days after inoculation parasites were maintaining their original developmental schedule (data not shown), and previous work suggests that if adjustment occurs, it takes at least 7 days [3–7].

Why might ring stage parasites suffer from schedule mismatch whereas trophozoite stages benefit? One explanation is that it is simply costly for parasites to enter the host in the evening (when mismatched ring stages and matched trophozoites were inoculated, Figure 3). Given that these costs are independent of the route of infection and that costs manifest between day 1–2 pi (when the IP-injected parasites have appeared in the blood) processes operating in the bloodstream are likely responsible. Many components of mammalian blood, including RBC [2, 8], and immune factors in the blood and spleen exhibit circadian periodicity and often appear to be upregulated in the dark phase of the day [9–17]. However, whether such responses would only impact on parasites in the first 1 or 2 days post infection is unknown. There may be immune responses that are short acting, upregulated in the dark phase, directed against parasites, and that can be overwhelmed above a threshold parasite density [18]. Or, an immune response that is only effective at low densities may only be active during the first few bouts of parasite replication (schizogony). Alternatively, if some immune response(s) are upregulated in the dark phase and directed towards anomalous RBC, then RBC from donor mice may be recognised and cleared by this process. In this case, once parasites have undergone schizogony they reside in the host’s own RBC and escape this process on all subsequent days.

**Figure 3:**
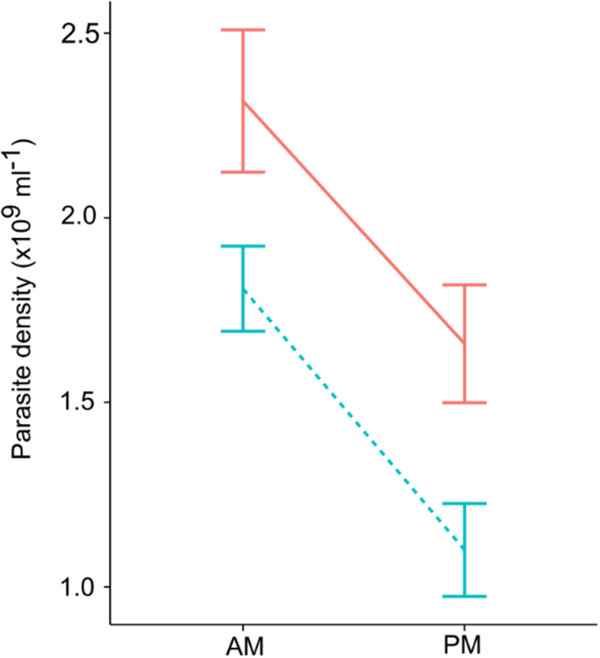
**Performance of parasites entering the host in the evening and morning.** Re-plot of data in Figure 1c showing mean (±se) densities achieved during the pre-peak phase of infections by different stages (rings, solid lines; trophozoites, dotted lines).

That the effect of schedule mismatch is not influenced by the route of infection (IP or IV) is unexpected. Macrophages line the peritoneal cavity and have an autonomous 24-hour clock that regulates phagocytosis and the rhythmic secretion of TNF and IL-6 in response to infection, with peak activity late in the day [9, 15, 17]. Parasites – at any stage - administered via IP in the evening were, therefore, expected to experience a harsher environment than parasites inoculated IP in the morning. Furthermore, late-stage parasites are thought to be more susceptible to stress than rings, as suggested for fever (e g, heat shock disproportionately kills parasites in the latter half of the cell cycle [19, 20]). If such stressors included active macrophages then trophozoites would be more vulnerable than rings when inoculated in the evening via IP. This is not the case because whilst trophozoites perform better when inoculated in the morning, this was not restricted to the IP group (i e, the 3-way interaction between schedule, stage, and route was not significant).

## Conclusions

It is beneficial for infections initiated with ring stage parasites to be in synchrony with their host’s rhythm and for trophozoites to be out of sync, regardless of the route of infection. The data presented here suggest mismatch impacts on the ability of ring stage parasites to establish infections, but not on their ability to multiply, and that the reduction in ‘starting number’ has a magnifying effect on density as infections progress. How different parasite stages are affected by synchronisation with the host, and why this is particularly important at the start of infections, also remains unknown. The answers to these questions may be revealed by directly testing whether parasite stages differ in their vulnerability to circadian innate effectors, if parasites have resource requirements that are only met at certain times of day, and how these processes are affected by parasite density. Unravelling the mechanisms that explain the differential effects of mismatch is necessary to determine whether the synchronicity and schedules of *P. chabaudi* cell cycles is under the control of parasites or hosts. Given that arrested cell-cycle development (quiescence) is implicated in tolerance to drugs [21–25], understanding what governs these schedules as well as the costs and benefits of adjusting them is important.
